# HCV Self-Testing to Expand Testing: A Pilot Among Men Who Have Sex With Men in China

**DOI:** 10.3389/fpubh.2022.903747

**Published:** 2022-05-31

**Authors:** Wenqian Xu, Elena Ivanova Reipold, Peizhen Zhao, Weiming Tang, Joseph D. Tucker, Jason J. Ong, Jinshen Wang, Philippa Easterbrook, Cheryl Case Johnson, Muhammad S. Jamil, Cheng Wang

**Affiliations:** ^1^School of Public Health, Southern Medical University, Guangzhou, China; ^2^Dermatology Hospital of Southern Medical University, Guangzhou, China; ^3^Southern Medical University Institute for Global Health, Guangzhou, China; ^4^Guangdong Provincial Center for Skin Diseases and STIs Control, Guangzhou, China; ^5^The Foundation for Innovative New Diagnostics, Geneva, Switzerland; ^6^University of North Carolina Project-China, Guangzhou, China; ^7^Faculty of Infectious and Tropical Diseases, London School of Hygiene and Tropical Medicine, London, United Kingdom; ^8^School of Medicine, Institute for Global Health and Infectious Diseases, University of North Carolina at Chapel Hill, Chapel Hill, NC, United States; ^9^Central Clinical School, Monash University, Melbourne, VIC, Australia; ^10^Department of Global HIV, Hepatitis, and STI Programmes, World Health Organization, Geneva, Switzerland

**Keywords:** hepatitis C virus, self-testing, men who have sex with men, rapid diagnostic tests, China

## Abstract

**Background:**

Hepatitis C virus self-testing (HCVST) may increase test uptake especially among marginalized key populations such as men who have sex with men (MSM). We conducted an observational study to assess the usability, acceptability and feasibility of HCVST among MSM in China.

**Methods:**

An observational study with convenience sampling was performed among MSM in Guangzhou, China in 2019. The OraQuick® HCV Rapid Antibody Test kits were used in this study. Participants performed all 12 HCVST steps and interpreted the results in the presence of a trained observer. Usability was defined as the number and percentage of participants who completed all testing steps correctly without assistance and interpreted the results correctly. Inter-reader concordance was calculated as the percentage agreement between the results interpreted by the participant and those interpreted by a trained staff member. The same process was used to estimate inter-operator agreement between the self-testing and professional use test results. Acceptability was assessed using an interviewer-administered semi-structured questionnaire.

**Results:**

Among 100 participants with median age 27 (interquartile range 23–30) years, 4% reported prior history of HCV testing, 41% reported using blood-based HIV self-testing in the past, 54% (95%CI: 43.7–64.0%) completed all self-testing steps correctly without assistance and interpreted the results correctly. Both the inter-reader and inter-operator concordance were excellent at 97% (95%CI: 91.5–99.4%) and 98% (95%CI: 93.0–99.8%), respectively. The majority rated the HCVST process as very easy (52%, 95%CI: 41.8–62.1%) or easy (41%, 95%CI: 31.3–51.3%), 76% (95%CI: 66.4–84.0%) were willing to use HCVST again, and 75% (95%CI: 65.3–83.1%) would recommend it to their family and friends.

**Conclusions:**

Our findings demonstrate that oral fluid HCVST has high usability and acceptability among Chinese MSM. More implementation research is needed to plan how best to position and scale-up HCVST alongside other facility-and community-based testing approaches and ensure data linkage into health systems.

## Introduction

Hepatitis C virus (HCV) infection is an urgent public health priority worldwide (1). Untreated HCV infection can lead to life-threatening complications of cirrhosis and hepatocellular carcinoma (2). World Health Organization (WHO) estimated that in 2019, approximately 58 million people were living with HCV globally (3). Despite the availability of low cost generic curative treatment for HCV, less than a quarter of those infected have been tested and aware of their infection status, and even lower in low- and middle-income countries (LMICs) (3). Men who have sex with men (MSM) are disproportionately affected by HCV infection, especially for MSM living with human immunodeficiency virus (HIV) (2, 4). A global systematic review and meta-analysis conducted in 2015 reported the overall HCV seroprevalence among MSM was 6.4% (3.2–10.0%) worldwide (5). In China, the estimated prevalence of HCV in MSM was 0.67% (6), but 8.4% among MSM living with HIV (7). A nationwide survey conducted in 2017 showed that only 41% of Chinese MSM had ever received an HCV test (8).

Many factors likely underpin low HCV test uptake among MSM in low- and middle-income countries. In many LMICs with generalized epidemics, due to unstable socioeconomic status (9) and lack of knowledge about HCV (10), members of marginalized populations may have a low self-perceived risk of infection. In addition, facility-based testing for HCV has potential limitations related to stigma from health care providers (11), infrastructure requirements, inconvenience, and lack of privacy (12). There is a need for additional strategies to promote uptake of HCV testing and linkage to care, and self-testing represents one such approach.

Self-testing is the process by which an individual collects their specimen, performs the test, and interprets the results by themselves (2). Self-testing diversifies testing locations (13), safeguards confidentiality (14) and simplifies the process of diagnostic testing (13). Self-testing may also decrease any stigma associated with testing in the clinic (14), as it provides an opportunity for key populations to test themselves discreetly and conveniently. HIV self-testing (7, 13, 15) and syphilis self-testing (16, 17) is now already adopted as a complement to facility-based testing services to improve test uptake among key populations. To introduce and promote HCV self-testing, in 2019, the Foundation for New Innovative Diagnostics (FIND) in collaboration with WHO, conducted a series of pilot studies of HCV self-testing in a range of different settings across five countries, including China. These findings led to the development of the first global HCV self-testing guidelines, which was published in 2021 by WHO, recommending HCV self-testing as an additional testing approach to increase coverage of those aware of their diagnosis (3). To inform implementation and scale-up decisions on HCV self-testing at a national level, we analyzed data from this observational study to assess the usability, acceptability and feasibility of HCV self-testing among MSM in China.

## Methods

### Setting and Population

The study was conducted at two sites in Guangzhou: an office of a local MSM community-based organization (CBO) and a hospital outpatient clinic at Guangdong provincial center for Sexually Transmitted Disease (STD) control. These sites and their services were selected for the pilot study because they were developed with input from MSM and offer free HIV and syphilis testing services. Both sites are staffed by peer MSM volunteers, nurses, and public health staff.

Eligible participants were men aged 18 years or above who identified as MSM and had at least one anal sex episode with another man within the last 6 months, but had no prior experience of oral fluid-based HIV and/or HCV self-testing; and were not known to have HCV infection (treated or untreated); were able to understand the scope of the study and provide written informed consent; and able to read in Mandarin Chinese.

### Sample Size

As there were no published data on HCV self-testing acceptability at the time of study design, we made a conservative assumption that 50% of eligible individuals would agree to perform a self-test. To estimate the proportion in this study with a 95% confidence interval based on Wilson's score method, with ±10% margin of error, we assumed a minimum sample size of 97 participants was required and rounded the resultant sample size up to the nearest 100.

### Test Kits

We used the OraQuick® HCV Rapid Antibody Test (OraSure Technologies Inc., Bethlehem, PA, USA) kits in this study. This test is a 20-min immunoassay for the qualitative detection of HCV antibodies in oral fluids, with a 99.4% sensitivity and 100% specificity (18). The OraQuick® HCV Rapid Antibody Test has been prequalified by WHO for professional use (19). For research, the test was repackaged by the manufacturer for self-testing. The package included one OraQuick® HCV Rapid Antibody Test, developer solution, a plastic test stand, desiccant and manufacturer instructions provided in Mandarin Chinese. As the OraQuick® HCV Self-Test is not approved for use in China, the test results were not used to make any clinical decisions and were for investigational use only.

### Study Procedures

From July to September 2019, potential participants seeking routine clinical testing and care were screened for eligibility and invited to participate by MSM volunteers working at the two recruitment sites. Eligible individuals who agreed to participate were provided with a written informed consent form and invited into a private room to complete all the study steps. Baseline information on demographic characteristics, risk factors for HCV infection and previous experience of HCV testing were collected first by the study staff. Study participants were provided with written instructions in Chinese on how to perform the HCV self-test, and were invited to complete all the study steps in the private room (Supplementary Figure S1). They were also asked to not eat or drink 15 mins prior to the testing procedure and to refrain from using oral care products 30 mins prior to testing. Participants were renumerated with $8 USD for their time and informed that their information would be kept confidential. All the study steps were administered by trained study staff.

The HCV self-testing process included 12 steps. Pre-testing steps were: opening the package; reading the instructions for use before or during testing; removing the test tube from the test pack; removing the cap from the test tube; sliding the test tube into the stand; removing the test device from the test pack. Performance steps were: handling the device correctly (i.e., not touching the flat pad used for specimen collection); collecting the sample; placing the test device in the test tube; and monitoring the time while waiting for the result. The final step was correct reading and interpretation of the test result.

Participants were asked to conduct the test and interpret the result by themselves while observed by study staff trained in the assessment of the HCV self-test procedure. The study staff documented if the testing was completed correctly and documented any observed mistakes using a standardized checklist. The staff were given specific instructions to not intervene and provide any assistance during the testing procedures, unless participants had exhausted all attempts to complete the testing steps (usually after 15 min of trying without success). Assistance provided by the staff was also documented using the checklist.

A post-test interview was conducted using a semi-structured questionnaire to assess participants' perception of acceptability and preference for HCV self-testing. The used self-test kits were provided to a staff member trained in interpreting the self-test results immediately after use for independent re-reading to measure inter-reader concordance. Results were not shared with the study participants to avoid bias in responses regarding the perception of HCV self-testing. After completion of the post-testing interview, a professional use rapid test was performed for each participant by another trained study staff blinded to the self-reported results. We used this second test result to compare with the self-tester's result to measure the inter-operator concordance.

After completing all study procedures, all participants received the standard blood-based HCV serology test routinely used at the study sites. Post-test counseling was provided to all study participants. Individuals with reactive results were informed as to the meaning of a positive result, the necessity of retesting in the health facility, and the location of the health facility where confirmatory tests, diagnosis and treatment could be performed in Guangzhou.

Routine data collected included Sociodemographic characteristics included age, gender, marital status, education level, and employment status. Behavioral characteristics included frequency of attending health facilities, HCV and HIV testing history, whether the individual had ever engaged in the following activities in their lifetime: condomless anal intercourse, injected drugs or shared needles with others, had a surgical procedure or a dental procedure, shared shaving tools or toothbrushes with others, or had tattoos.

### Analysis

#### Usability

Usability was defined as the number and percentage of participants who completed all testing steps correctly without assistance and interpreted the results correctly. Information on errors, difficulties and assistance needed at each step of the testing procedure was recorded. Categories of testing results were defined as positive, negative or invalid.

#### Inter-reader Concordance and Inter-operator Concordance

Inter-reader concordance was defined as the agreement between the results reported by the participant and re-read by a study staff member, reported as a percentage. Inter-operator concordance was defined as agreement between the results of self-testing reported by the participant and results of the professional use rapid test conducted by a study staff member, reported as a percentage.

#### Acceptability and Feasibility

Acceptability included pre-test and post-test acceptability. Pre-test acceptability was defined as the proportion of individuals who agreed to participate in the study and perform self-testing among the total number of eligible participants. Post-test acceptability was defined as the proportion of study participants who stated that they would use the HCV self-test again and recommend it to family or friends. Feasibility included the following: whether the participant rated the instructions as easy to understand, participant's perspectives of the HCV self-testing at different steps, follow-up actions after receiving a positive/reactive result, and experiences throughout the testing process.

Analyses were performed using IBM SPSS Statistics (version 23). The results were reported as a percentage (95% confidence interval) or median (interquartile range) as appropriate. The usability, including errors, difficulties and requirements for assistance recorded during the self-testing procedure, was reported using descriptive statistics. Both inter-reader concordance and inter-operator concordance was reported as a percentage (95% confidence interval). Cohen's kappa coefficient was calculated in two ways: one including invalid results and one excluding invalid result. Acceptability and feasibility were presented using descriptive statistics.

### Ethics Review

This study was approved by the Office of Ethical Review Committee of Dermatology Hospital of Southern Medical University (Document No: GDDHLS-20190307). Written informed consent was obtained from all the participants who agreed to participate in this study. Each participant was allowed to sign informed consent using pseudonym to assure anonymity. The study was conducted in accordance with the ethical principles derived from international guidelines including the Declaration of Helsinki, and with applicable laws and regulations.

## Results

### Participant Characteristics

**Figure 1:**
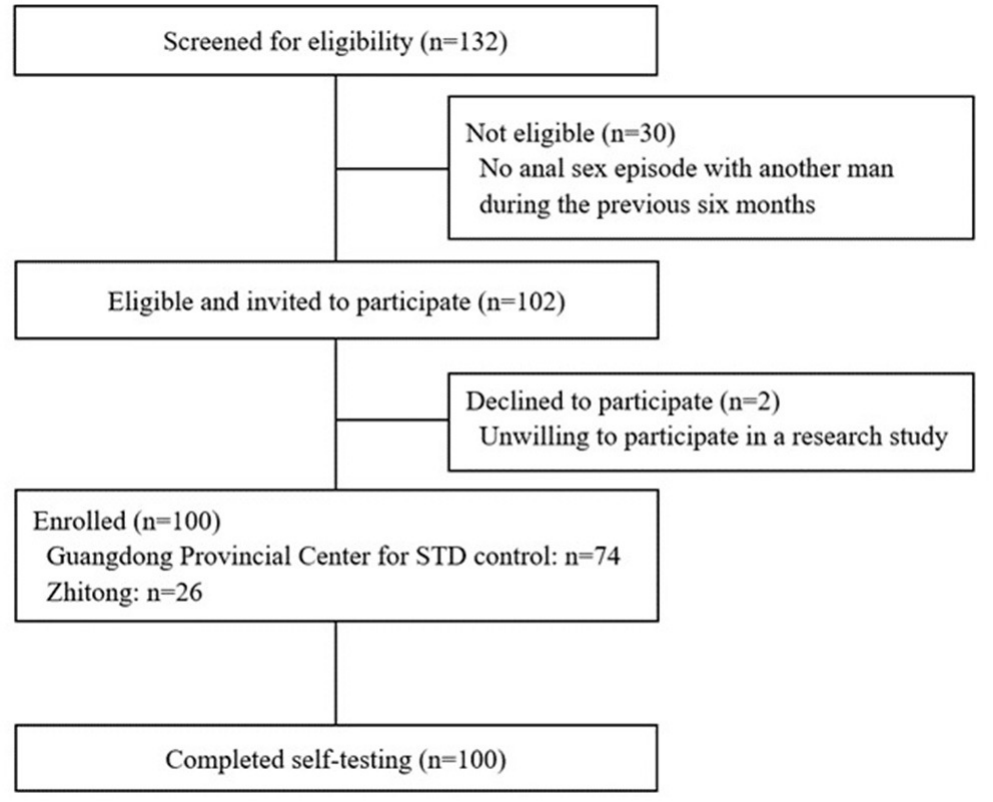
Flowchart of the study population.

Overall, 132 individuals were screened for eligibility. Of these, 30 did not meet eligibility requirements (did not engage in anal sex with a man within the previous 6 months), and two did not sign the consent form. A hundred men were enrolled in the study, of whom 74 were enrolled at the Guangdong Provincial Center for STD control and 26 from the Zhitong office (Figure 1).

The median age of the participants was 27 years (IQR: 23–30). The majority were unmarried (89%), had a bachelor's degree or higher (51%), were employed (79%), and had engaged in condomless anal intercourse (57%). Only one participant reported injecting unprescribed drugs or sharing needles. Four had previously tested for HCV, and 90 for HIV. Almost all participants at baseline were aware of the existence of self-testing (92% stated that they were aware that certain tests could be performed at home), and 41% had used blood-based HIV self-tests in the past (Table 1).

**Table 1:** Baseline demographic characteristics among MSM, an observational study in China, 2019, *N* = 100.

**Characteristics**	***n***	**%**
Median age, years (IQR)	27 (23–30)	
Sex		
Male	99	99
Transgender	1	1
Marital status		
Ever married	11	11
Never married	89	89
Educational background		
High school or below	25	25
Junior college	24	24
Bachelor's degree and above	51	51
Employment status		
Unemployed	21	21
Employed	79	79
Frequency of routine health checks		
More than one time per year	42	42
One time per year	41	41
Rarely (once in two or more years)	11	11
Never	6	6
Self-reported exposure (ever) to any of the following risk factors for HCV infection		
Condomless anal intercourse	57	57
A surgical procedure	26	26
A dental procedure	28	28
Sharing shaving tools or toothbrushes	15	15
Make a tattoo	5	5
Injecting unprescribed drugs or sharing needles	1	1
None of listed above	21	21
Ever had HCV testing	4	4
Ever had HIV testing	90	90
Ever had HIV self-testing	41	41
Sex partner ever tested for HCV	2	2
Sex partner ever tested for HIV	33	33
Awareness of self-testing		
Aware that certain kinds of tests can be performed at home	92	92
Ready to use HCV self-testing if available	93	93

*IQR, interquartile range; HCV, hepatitis C virus; HIV, human immunodeficiency virus*.

### Usability of HCV Self-Testing

Overall, 96 participants were able to complete the HCV self-test, and 54% (95%CI: 43.7–64.0%) completed all steps correctly without assistance and interpreted the results correctly. Forty-four percent made an error during at least one testing step (excluding one participant who misinterpreted the result and another who required assistance). Four steps were conducted correctly by all participants, including opening the pouch, reading the instructions during the testing, removing the test tube from the test pack, and removing the cap from the test tube. The most frequently observed errors were not using a time-keeping device (19%, 95%CI: 11.8–28.1%), not reading the results between 20 and 40 mins (18%, 95%CI: 11.0–26.9%), and touching the flat pad (15%, 95%CI: 8.6–23.5%). In addition, a small proportion collected samples incorrectly (9%, 95%CI: 4.2–16.4%), and had difficulty placing the test device in the test tube (3%, 95%CI: 0.6–8.5%). The majority of participants (97%, 95%CI: 91.5–99.4%) interpreted the test results correctly. The most frequently observed difficulty was sliding the tube into the stand (35%, 95%CI: 25.7–45.2%), followed by opening the tube (32%, 95%CI: 23.0–42.1%). Assistance was provided to a minority of individuals when sliding the tube into the stand (2%, 95%CI: 0.2–7.0%) and reading the results (2%, 95%CI: 0.2–7.0%) (Table 2).

**Table 2:** Observer assessment of successful steps, difficulties, and steps requiring assistance among MSM, an observational study in China, 2019.

**Testing steps**	***n***	***%(95%CI)***
**Observed errors at each step using the usability checklist**		
Pretesting		
Opening the pouch	0	0.0 (0.0–3.6)
Reading/using the instructions for use before the testing	1	1.0 (0.0–5.4)
Reading/using the instructions for use during the testing	0	0.0 (0.0–3.6)
Removing the test tube from the test pack	0	0.0 (0.0–3.6)
Removing the cap from the test tube	0	0.0 (0.0–3.6)
Sliding the test tube into the stand	3	3.0 (0.6–8.5)
Removing the test device from the test pack	1	1.0 (0.0–5.4)
Conducting the test		
Not touching the flat pad	15	15.0 (8.6–23.5)
Incorrectly collecting the sample	9	9.0 (4.2–16.4)
Incorrectly placing the test device in the test tube	3	3.0 (0.6–8.5)
Using a time keeping device	19	19.0 (11.8–28.1)
Reading the device results between 20 and 40 min	18	18.0 (11.0–26.9)
**Difficulties** ^†^		
Opening the pouch	2	2.0 (0.2–7.0)
Placing the test device into the tube	3	3.0 (0.6–8.5)
Reading the results	7	7.0 (2.9–13.9)
Opening the tube	32	32.0 (23.0–42.1)
Sliding the tube into the stand	35	35.0 (25.7–45.2)
**Assistance** ^**‡**^		
Reading the results	2	2.0 (0.2–7.0)
Sliding the tube into the stand	2	2.0 (0.2–7.0)
**Test interpretation**		
Interpreted test results incorrectly (result read by the study participant was not in agreement with re-reading by the trained staff member)	3	3.0 (0.6–8.5)
**Completed all testing steps correctly without any assistance and interpreted the test results correctly**	54	54.0 (43.7–64.0)

*CI, confidence interval; HCV, hepatitis C virus*.

*The most difficult part of the self-testing that study staff observed*.

*Assistance was provided by the observers*.

### Interpretation of Self-Test Results

Among the 100 participants, 96 self-reported that their test results were negative, two that results were invalid, and two self-reported uncertain results. No participants self-reported a positive result. Inter-reader concordance between the participants' self-reported results and the researchers' re-read was 97% (95%CI: 91.5–99.4%). Among the three cases with discordant results, one was interpreted as negative by the participant but was deemed invalid by the staff member. This participant made two errors (swabbed only the upper gums, touched the flat pad), requested assistance with sliding the test tube into the stand, and had an education level below high school. For the remaining two cases, the participants could not interpret their results. The study staff found one of these had a negative result, and the participant had made some mistakes (poured the liquid into the holder, put the test equipment on the holder, and then read the test results) and reported the testing process to be “somewhat difficult.” Another case was interpreted as invalid by the staff member; this participant had experienced difficulties placing the test tube into the stand and requested assistance with reading the results (Supplementary Table S2).

For the measure of inter-operator concordance, all results were negative when the study staff retested the 100 participants, and the concordance of results between “self-testing” and “retesting” was 98% (95%CI: 93.0–99.8%) [excluding invalid results (*n* = 2)]. According to the professional use test, the two results of which the participants were unsure were HCV negative (Table 3).

**Table 3:** Assessment of inter-reader and inter-operator concordance among MSM, an observational study in China, 2019.

		**Re-reading by a staff member** ^**†**^				**Retesting by a staff member** ^**‡**^			
		**Positive**	**Negative**	**Invalid**	**Unsure**	**Positive**	**Negative**	**Invalid**	**Unsure**
Self-reported	Positive (*n* = 0)	0	0	0	0	0	0	0	0
	Negative (*n* = 96)	0	95	1	0	0	96	0	0
	Invalid (*n* = 2)	0	0	2	0	0	2	0	0
	Unsure (*n* = 2)	0	1	1	0	0	2	0	0
Concordance (%)		Between self-testing and rereading 97% (95%CI: 91.5–99.4)				Between self-testing and retesting 98%^§^ (95%CI: 93.0–99.8)			

*CI, confidence interval*.

*^†^The participant's self-testing results were re-read and explained by a staff member*.

*^‡^The results of the second test conducted by the staff using the oral fluid OraQuick®HCV rapid antibody test*.

*§Excluding invalid results*.

### Acceptability and Feasibility

Prior to taking the test, 94% (95%CI: 87.4–97.8%) of the 100 study participants expressed a willingness to use HCV self-testing if available. Post-testing acceptability was 76% (95%CI: 66.4–84.0%), most stated they would use the HCV self-test again and recommend it to family or friends (Table 4). Reasons given for using HCV self-testing again if available included provision of information about personal health by regular testing (27%, 95%CI: 18.6–36.8%), convenience (17%, 95%CI: 10.2–25.8%), and simplicity (7%, 95%CI: 2.9–13.9%). Reasons for not using HCV self-testing again included lack of need (3%, 95%CI: 0.6–8.5%), preference for facility-based testing (2%, 95%CI: 0.2–7.0%) and lack of knowledge about HCV (1%, 95%CI: 0.0–5.4%). Reasons for being unsure about using HCV self-testing were lack of information about the test (5%, 95%CI: 1.6–11.3%) and lack of awareness about risk of HCV infection (2%, 95%CI: 0.2–7.0%) (Supplementary Table S3).

**Table 4:** Participant views and preferences regarding HCVST, an observational study in China, 2019.

**Acceptability**	***n***	***%(95%CI)***
**Before self-testing**		
The proportion of participants among eligible individuals who agreed to participate and perform HCVST	100/102	98.0 (93.1–99.8)
Ready to use HCV self-test if available	94/100	94.0 (87.4–97.8)
**After self-testing (*****N*** **=** **100)**		
Willing to use HCV test again	76	76.0 (66.4–84.0)
Willing to recommend the test to family and friends	75	75.0 (65.3–83.1)
Taking the tests to family member/friend	73	73.0 (63.2–81.4)
**Preferences with regard to HCVST**	***n***	***%(95%CI)***
Preferred approach to test for HCV in the future (*N* = 100)		
By myself at a healthcare facility	18	18.0 (11.0–26.9)
In a screening campaign	18	18.0 (11.0–26.9)
Taking a regular sample at a healthcare facility	35	35.0 (25.7–45.2)
In a healthcare facility by healthcare worker	41	41.0 (31.3–51.3)
By myself at home	52	52.0 (41.8–62.1)
Preferred sample type (*N* = 100)		
Prefer blood-based test	19	19.0 (11.8–28.1)
No preference	22	22.0 (14.3–31.4)
Prefer oral fluid-based test	59	59.0 (48.7–68.7)
What to do if positive (*N* = 100)		
Do not know	0	0.0 (0.0–3.6)
Contact pharmacy	10	10.0 (4.9–17.6)
Seek advice from a community representative (e.g., NGO representative)	22	22.0 (14.3–31.4)
Seek advice from family members and/or friends	25	25.0 (16.9–34.7)
Do a confirmation test (viral load test)	78	78.0 (68.6–85.7)
Contact health facility	83	83.0 (74.2–89.8)
Knowledge about HCV treatment (*N* = 100)		
There is no treatment or cure	1	1.0 (0.0–5.4)
Not sure if there is treatment	10	10.0 (4.9–17.6)
Know that there is a treatment but not sure about the cure	21	21.0 (13.5–30.3)
Know that HCV can be cured	21	21.0 (13.5–30.3)
No idea	47	47.0 (36.9–57.2)

*CI, confidence interval; HCV, hepatitis C virus; HCVST, HCV self-testing*.

Overall, 75% (95%CI: 65.3–83.1%) reported they would recommend the test to family and friends (Table 4). The most common reasons for recommending the test were convenience (30%, 95%CI: 21.2–40.0%), followed by the provision of information about personal health (22%, 95%CI: 14.3–31.4%). The most common reasons for not recommending the test were lack of knowledge about HCV (4%, 95%CI: 1.1–9.9%), followed by lack of need (3%, 95%CI: 0.6–8.5%). The most common reasons for being unsure about recommending the test were lack of confidence in the results (6%, 95%CI: 2.2–12.6%) or lack of knowledge about HCV (2%, 95%CI: 0.2–7.0%) (Supplementary Table S3).

Approximately 52% (95%CI: 41.8–62.1%) of participants reported that their preferred approach for HCV testing in the future would be self-testing at home, while 41% (95%CI: 31.3–51.3%) would go to a healthcare facility for testing by the healthcare worker. There was a preference for oral fluid-based HCV self-testing in 59% (95%CI: 48.7–68.7%) and blood-based testing in 19% (95%CI: 11.8–28.1%). The majority (83%, 95%CI: 74.2–89.8%) stated that they would contact a health facility following positive HCV self-test result; around a third (25%, 95%CI: 16.9–34.7%) said that they would seek advice from a family or community member. Knowledge about HCV treatment was poor in participants; only 21% (95%CI: 13.5–30.3%) were aware that HCV could be cured, and a further 21% (95%CI: 13.5–30.3%) knew that there was a treatment but were unsure if it was a cure (Table 4). The majority (78%, 95%CI: 68.6–85.7%) were unsure or had no idea whether treatment was available for HCV in their setting (Supplementary Table S3).

**Figure 2:**
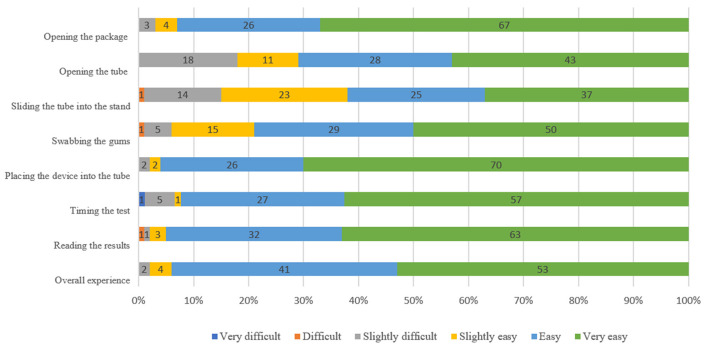
Participants' perceptions of HCVST usability at different steps among MSM, an observational study in China, 2019.

The majority of participants (94%, 95%CI: 11.8–28.1%) rated the steps as “very easy,” “easy” or “slightly easy” (Figure 2). The steps perceived as being most difficult were opening the tube [rated as “slightly difficult” by 18% (95%CI: 11.0–26.9%)] and sliding the tube into the stand [rated as “slightly difficult” by 14% (95%CI: 7.9–22.4%) and “difficult” by 1% (95%CI: 0.0–5.4%)] (Figure 2). Overall, 93% (95%CI: 86.1–97.1%) were “very satisfied” or “somewhat satisfied” with the HCV self-testing process. Advantages of the HCV self-testing reported by the participants included the flexibility to test at any time (90%, 95%CI: 82.4–95.1%), no need to go to a clinic (78%, 95%CI: 68.6–85.7%), and privacy (61%, 95%CI: 50.7–70.6%). Disadvantages included lack of counseling when receiving results (21%, 95%CI: 13.5–30.3%) and lack of confidence in test results (20%, 95%CI: 12.7–29.2%); however, 96% (95%CI: 90.1–98.9%) stated that they would be comfortable reading an HCV self-test result alone. In total, 35% (95%CI: 25.7–45.2%) stated that there were no disadvantages to HCV self-testing (Supplementary Table S3).

## Discussion

MSM are at high risk of HCV acquisition and transmission (1). Our study showed high acceptability and moderate usability of the oral HCV rapid antibody test as a self-test among Chinese MSM. This study expands the literature by examining the usability, feasibility, and acceptability of HCV self-testing in China. Similar pilot studies on HCV self-testing have been conducted by the FIND and WHO team in different settings and populations in Vietnam (20), Egypt (21), Kenya (22) and Georgia. Findings from this study provide insights for the implementation of HCV self-testing programmes and research among MSM.

Our data suggested moderate usability of the HCV self-test among Chinese MSM. Just over half of 100 self-testers were able to complete all steps of the HCV self-test, and almost all interpreted the results correctly (most without assistance). We found the most frequently observed difficulties related to sliding the tube into the stand and removing the cap from the test tube, consistent with studies in Egypt (21) and Vietnam (20). We also found that the steps at which errors were observed differed slightly between the studies in China, Egypt (21) and Vietnam (20). Although incorrect timekeeping (where users failed to wait the required 15 mins for the test result) and touching the pad used for specimen collection were frequently observed in China and Egypt, incorrect placement of the tube into the stand was more frequent in our study (9% vs. 0.8%), while fewer participants from Egypt were able to correctly interpret the test results (97% vs. 86%) (21). Lower error rates were observed in the pilot study in Vietnam, in which the most common mistakes made by MSM were incorrect collection of the oral fluid sample (14%) and touching the flat pad (4%) (20). These differences across the three studies may be due to instruction issues and thus may be resolvable by amending the manufacturer's instructions for use (such as highlighting important contents or adding patterns of tips) or clearer labeling on the test kit components. Alternatively, these errors may arise from cultural differences across countries and populations studied, which need to be considered in the translation of instructions for use. In addition, only 4% asked for assistance in our study, in contrast to the experience from Vietnam, where 17.3% of MSM asked for assistance (20) and Egypt (12% of the general population asked for assistance) (21). The lower proportion requiring assistance may be due to MSM in our study already being familiar with self-testing in the context of HIV and syphilis.

In early studies of HIV self-test kits, usability was lower (85% failed to perform correctly), and user errors and difficulties with different testing steps were frequent (23). However, it is encouraging to note that usability of HIV oral fluid-based self-tests reported in recent studies has improved (24–28), with lower error rates (10%) (26) and fewer individuals requiring assistance (10–18%) (27). This provides confidence that greater optimisation of the HCV kit for self-testing is likely to improve overall usability.

In this study, we found good inter-reader concordance, with a high proportion of participants able to correctly interpret the HCV self-test results. The inter-reader concordance was higher than that observed in the pilot study in Egypt (97% vs. 86%) (21), but similar to that observed in MSM in Vietnam (97% vs. 99%) (20), and within the range observed across the majority of studies of HIV self-tests (85.4–100%) (23). A prior HIV self-test study in Zimbabwe reported that lower inter-reader concordance was attributed to low literacy and verbose instructions for use (23). In another study in MSM from China, the accuracy of performance of an HIV self-test was associated with education level (29). The younger age and higher education level of most participants in our study may have contributed to the high inter-reader concordance. There may be a need for additional support tools in certain populations, such as older, less educated, or those with issues reading or understanding instructions. Instructional videos, mobile apps or virtual assistance could be helpful in these cases. Our data suggest a high inter-operator concordance, demonstrating that the self-test kit provides consistent results with the professional-use version.

We found a high level of acceptability of HCV self-testing among Chinese MSM, consistent with reports for HCV self-testing from FIND projects in Vietnam (20), Egypt (21), Kenya (22), and Georgia as well as one other small pilot study in the UK (30). After the self-testing experience, approximately three-quarters of participants expressed a willingness to use an HCV self-test again or to recommend the self-test to family and friends. The advantages of HCV self-test reported by participants included great privacy, convenience and the ability to decide when and how to seek treatment and care, suggesting that HCV self-testing may remove barriers linked with traditional HCV testing and facilitate testing. Meanwhile, our study found that post-test acceptability of HCV self-testing in this MSM population was lower than that observed among MSM in Vietnam (20) and in the general population in Egypt (21). Six participants in our study stated that they would not use HCV self-testing again because of preference for facility-based testing and/or lack of need. The preference for facility-based testing may be related to the fact that our study sites were located in a city setting where there was ready availability of facility-based testing. Individuals from rural or remote settings have difficulties accessing facility-based services, so they may derive more benefit from self-testing options. It is possible that participants who cited a lack of need as a reason were unaware that they were at high risk or had low incentive to seek testing due to a perceived lack of treatment. Meanwhile, our data suggest that knowledge about HCV among participants in this study was poor. This demonstrates a need for enhanced education in high-risk populations to raise awareness of those infections for which they are at risk and their prevention.

This study has implications for research and implementation. First, there is a need to optimize the instructions, improve the test kits, and provide support tools to make HCV self-testing more user-friendly. Actions that can be taken include simplifying packaging, clearly marking kit components, placing particular emphasis on key steps in instructions where participating MSMs were found to have the most difficulty in, offering educational pictogram or video demonstrations. Second, we found that the cost of HCV self-testing can be an important barrier to its implementation. This suggests that subsidized or partially subsidized self-testing should be considered. Third, HCV self-testing could be used during COVID-19 measures. Although our study was conducted prior to COVID-19, closures in facilities during COVID-19 have accelerated the development and acceptability of self-testing approaches. Fourth, our study expands the evidence base for self-care interventions and would provide valuable data for self-care programs and research. HCV self-testing might play an important role in reducing undiagnosed infections and improving access to care services (31). Researches are required to inform linkage to clinical services, and how best to promote this within the context of larger-scale implementation.

This study has several limitations. First, participants were asked to perform only oral HCV tests and experience with blood-based self-testing was not assessed. Second, HCV self-testing is intended to be used privately, but participants were observed during the testing could have influenced their responses. Third, the HCV self-test kits used in this study were only for research use, and the packaging and instructions for use may be different from the final product. Further, the study included a convenience sample of MSM population in two study sites in Guangzhou, likely resulting in selection bias. This may overestimate usability and acceptability due to differences in health-seeking behaviors and the self-testing experience of clinic attendees compared with men who are less likely to attend these sites.

Notably, results from this study and the other pilot studies in Egypt and Vietnam using the same protocol can only evaluate usability and acceptability of the OraQuick® HCV Self-Test. More studies are needed to assess usability and acceptability of blood-based tests, as well as other oral fluid-based HCV self-tests, across different populations and settings, and to evaluate other aspects of HCV self-testing, including test performance, the impact on linkage to confirmatory diagnosis and treatment, and the need for support tools for certain populations.

In conclusion, our study demonstrated that oral fluid HCV self-testing is feasible and has high acceptability among Chinese MSM. Given the moderate usability and the potential of HCVST to remove barriers to facility-based HCV testing, this testing method may represent an option for reaching men and helping expand HCV testing.

## Data Availability Statement

The raw data supporting the conclusions of this article will be made available by the authors, without undue reservation.

## Ethics Statement

The studies involving human participants were reviewed and approved by Office of Ethical Review Committee of Dermatology Hospital of Southern Medical University. The patients/participants provided their written informed consent to participate in this study.

## Author Contributions

ER and PE designed and conceptualized the study. ER developed the protocol and data collection tools. CJ and PE critically reviewed the protocol and study tools. CW coordinated study implementation. WX and CW drafted the manuscript. WX conducted the statistical analysis. All authors were involved in the drafting and reviewing of the manuscript and approved the final version.

## Funding

This work was supported by the Foundation for Innovative New Diagnostics (HC19-0016).

## Conflict of Interest

The authors declare that the research was conducted in the absence of any commercial or financial relationships that could be construed as a potential conflict of interest.

## Publisher's Note

All claims expressed in this article are solely those of the authors and do not necessarily represent those of their affiliated organizations, or those of the publisher, the editors and the reviewers. Any product that may be evaluated in this article, or claim that may be made by its manufacturer, is not guaranteed or endorsed by the publisher.

## References

[1] World Health Organization. Accelerating Access to Hepatitis C Diagnostics and Treatment: Overcoming Barriers in Low- and Middle-Income Countries: Global Progress Report 2020. (2021). Available online at: https://apps.who.int/iris/handle/10665/338901 (accessed October 20, 2021).

[2] World Health Organization. WHO Guidelines on Hepatitis B and C Testing. (2017). Available online at: https://apps.who.int/iris/handle/10665/254621 (accessed August 20, 2021).

[3] World Health Organization. Recommendations and Guidance on Hepatitis C Virus Self-Testing. (2021). Available online at: https://apps.who.int/iris/handle/10665/342803 (accessed August 20, 2021).

[4] Jin FY Dore GJ Matthews G Luhmann N Macdonald V Bajis S. Prevalence and incidence of hepatitis C virus infection in men who have sex with men: a systematic review and meta-analysis. Lancet Gastroenterol Hepatol. (2021) 6:39–56. 10.1016/S2468-1253(20)30303-4 33217341

[5] Platt L Easterbrook P Gower E Mcdonald B Sabin K Mcgowan C. Prevalence and burden of HCV co-infection in people living with HIV: a global systematic review and meta-analysis. Lancet Infect Dis. (2016) 16:797–808. 10.1016/S1473-3099(15)00485-5 26922272

[6] Liu CR Li X Chan PL Zhuang H Jia JD Wang X. Prevalence of hepatitis C virus infection among key populations in China: a systematic review. Int J Infect Dis. (2019) 80:16–27. 10.1016/j.ijid.2018.11.006 30529371

[7] Chow EPF Tucker JD Wong FY Nehl EJ Wang YJ Zhuang X. Disparities and risks of sexually transmissible infections among men who have sex with men in China: a meta-analysis and data synthesis. PLoS ONE. (2014) 9:13. 10.1371/journal.pone.0089959 24587152 PMC3933676

[8] Fitzpatrick T Pan SW Tang W Guo W Tucker JD. HBV and HCV test uptake and correlates among men who have sex with men in China: a nationwide cross-sectional online survey. Sex Transm Infect. (2018) 94:502–7. 10.1136/sextrans-2018-053549 29779005 PMC6195464

[9] Kannan A Biswas L Kumar A Kurian J Nair AS Suresh P. Improving diagnosis of hepatitis C virus infection using hepatitis C core antigen testing in a resource-poor setting. Rev Soc Bras Med Trop. (2021) 54:e02532020. 10.1590/0037-8682-0253-2020 33605377 PMC7891558

[10] Lakoh S Garcia-Tardon N Adekanmbi O Van Der Valk M Smith SJ Grobusch MP. Prevalence of viral hepatitis B and C in Sierra Leone-current knowledge and knowledge gaps: a narrative review. Trans R Soc Trop Med Hyg. (2021) 115:1106–13. 10.1093/trstmh/trab054 33772308 PMC8486739

[11] Song Y Li X Zhang L Fang X Lin X Liu Y. HIV-testing behavior among young migrant men who have sex with men (MSM) in Beijing, China. AIDS Care. (2011) 23:179–86. 10.1080/09540121.2010.487088 21259130 PMC3076143

[12] Wang R Cui N Long M Mu L Zeng H. Barriers to uptake of hepatitis C virus (HCV) health intervention among men who have sex with men in Southwest China: a qualitative study. Health Soc Care Commun. (2021) 29:445–52. 10.1111/hsc.13104 32667104

[13] Kpokiri EE Marley G Tang W Fongwen N Wu D Berendes S. Diagnostic infectious diseases testing outside clinics: a global systematic review and meta-analysis. Open Forum Infect Dis. (2020) 7:ofaa360. 10.1093/ofid/ofaa360 33072806 PMC7545117

[14] Tucker JD Wei C Pendse R Ying-Ru L. HIV self-testing among key populations: an implementation science approach to evaluating self-testing. J Virus Erad. (2015) 1:38–42. 10.1016/S2055-6640(20)31145-6 26005717 PMC4439005

[15] Majam M Fischer A Reipold EI Rhagnath N Msolomba V Lalla-Edward ST. A lay-user assessment of hepatitis C virus self-testing device usability and interpretation in Johannesburg, South Africa. Diagnostics. 11:14. 10.3390/diagnostics11030463 33800060 PMC8000311

[16] Wang C Cheng W Li C Tang W Ong JJ Smith MK. Syphilis self-testing: a nationwide pragmatic study among men who have sex with men in China. Clin Infect Dis. (2020) 70:2178–86. 10.1093/cid/ciz603 31260513 PMC7201417

[17] Wu D Zhou Y Yang N Huang S He X Tucker J. Social media-based secondary distribution of human immunodeficiency virus/syphilis self-testing among Chinese men who have sex with men. Clin Infect Dis. (2021) 73:e2251–7. 10.1093/cid/ciaa825 32588883 PMC8492201

[18] Chevaliez S Poiteau L Rosa I Soulier A Roudot-Thoraval F Laperche S. Prospective assessment of rapid diagnostic tests for the detection of antibodies to hepatitis C virus, a tool for improving access to care. Clin Microbiol Infect. (2016) 22:6. 10.1016/j.cmi.2016.01.009 26806260

[19] World Health Organization. Prequalification of In Vitro Diagnostics. Public report. Product: OraQuick HCV Rapid Antibody Test Kit. (2021). Available online at: https://www.who.int/diagnostics_laboratory/evaluations/pq-list/hcv/170301_final_pq_report_PQDx_0244_055_00.pdfWorld (accessed August 20, 2021).

[20] Nguyen LT Nguyen VTT Le Ai KA Truong MB Tran TTM Jamil MS. Acceptability and usability of HCV self-testing in high risk populations in Vietnam. Diagnostics. (2021) 11:377. 10.3390/diagnostics11020377 33672241 PMC7926709

[21] Reipold EI Farahat A Elbeeh A Soliman R Aza EB Jamil MS. Usability and acceptability of self-testing for hepatitis C virus infection among the general population in the Nile Delta region of Egypt. BMC Public Health. (2021) 21:1188. 10.1186/s12889-021-11169-x 34158006 PMC8218412

[22] Reipold EI Fajardo E Juma E Bukusi D Aza EB Jamil MS. Usability and acceptability of oral fluid hepatitis C self-testing among people who inject drugs in coastal Kenya: a cross-sectional pilot study. Research Square. (2021). 10.21203/rs.3.rs-1128332/v1 PMC9479404 36109704

[23] Lee VJ Tan SC Earnest A Seong PS Tan HH Leo YS. User acceptability and feasibility of self-testing with HIV rapid tests. JAIDS. (2007) 45:449–53. 10.1097/QAI.0b013e318095a3f3 17554213

[24] Figueroa C Johnson C Ford N Sands A Dalal S Meurant R. Reliability of HIV rapid diagnostic tests for self-testing compared with testing by health-care workers: a systematic review and meta-analysis. Lancet HIV. (2018) 5:E277–90. 10.1016/S2352-3018(18)30044-4 29703707 PMC5986793

[25] Majam M Mazzola L Rhagnath N Lalla-Edward ST Mahomed R Venter WDF. Usability assessment of seven HIV self-test devices conducted with lay-users in Johannesburg, South Africa. PLoS ONE. (2020) 15:17. 10.1371/journal.pone.0227198 31935228 PMC6959591

[26] Choko AT Desmond N Webb EL Chavula K Napierala-Mavedzenge S Gaydos CA. The uptake and accuracy of oral kits for HIV self-testing in high HIV prevalence setting: a cross-sectional feasibility study in Blantyre, Malawi. PLoS Med. (2011) 8:11. 10.1371/journal.pmed.1001102 21990966 PMC3186813

[27] Sarkar A Mburu G Shivkumar PV Sharma P Campbell F Behera J. Feasibility of supervised self-testing using an oral fluid-based HIV rapid testing method: a cross-sectional, mixed method study among pregnant women in rural India. J Int AIDS Soc. (2016) 19:11. 10.7448/IAS.19.1.20993 27630096 PMC5023853

[28] Peck Roger B Lim Jeanette M Van Rooyen H Mukoma W Chepuka L Bansil P. What should the ideal HIV self-test look like? A usability study of test prototypes in unsupervised HIV self-testing in Kenya, Malawi, and South Africa. AIDS Behav. (2014) 18 Suppl 4:S422–32. 10.1007/s10461-014-0818-8 24947852

[29] Li Y Wang Y Zhang R Wang J Li Z Wang L. Analysis on accuracy and influencing factors of oral fluid-based rapid HIV self-testing among men who have sex with men. Zhonghua Liu Xing Bing Xue Za Zhi. (2016) 37:72–5. 10.3760/cma.j.issn.0254-6450.2016.01.015 26822647

[30] Guise A Witzel TC Mandal S Sabin C Rhodes T Nardone A. A qualitative assessment of the acceptability of hepatitis C remote self-testing and self-sampling amongst people who use drugs in London, UK. BMC Infect Dis. (2018) 18:8. 10.1186/s12879-018-3185-7 29914381 PMC6006927

[31] Peeling RW Boeras DI Marinucci F Easterbrook P. The future of viral hepatitis testing: innovations in testing technologies and approaches. BMC Infect Dis. (2017) 17:699. 10.1186/s12879-017-2775-0 29143676 PMC5688478

