# Supplementary material for: HCV Self-Testing to Expand Testing: A Pilot Among Men Who Have Sex With Men in China
Source: Front Public Health. 2022 May 31;10:903747. doi: 10.3389/fpubh.2022.903747 (PMC9194083; doi:10.3389/fpubh.2022.903747)
Supplement: Supplementary file 1 [file Data_Sheet_1.pdf]

Supplementary Material

Supporting Table S1. Data collection forms used in the study

Supporting Figure S1. Manufacturer's instructions for use (Mandarin Chinese)

Supporting Table S2. Discordances in results reported by participants and results obtained by re-reading and re-testing

Supporting Table S3. Additional Perceptions on HCV Self-Testing

Supporting Table S1. Data collection forms used in the study

Screening log

Site: ☐ Guangdong provincial center for Skin and STD control ☐ Office of Zhitong PI:

Nr.	Screening date (DD-MMM-YY)	Eligibility	Eligible?	Agreed to participate?	Reason if not agreed
	☐☐-☐☐☐-☐☐	☐ Age ≥ 18 years ☐ Male<br>☐ Able to understand the scope of the study and provide written informed consent<br>☐ Unknown HCV serology status<br>☐ Able to read in Mandarin Chinese<br>☐ Had at least one anal sex episode with another man within the previous 6 months<br>☐ Never used an oral-fluid based self-test	• YES<br>• NO	• YES<br>• NO	• Do not have time to participate in the study<br>• Do not want to participate in a research study<br>• Prefer to test elsewhere<br>• Prefer not to use self-test<br>• Prefer not to say<br>• Other: ___________________________
	☐☐-☐☐☐-☐☐	☐ Age ≥ 18 years ☐ Male	• YES<br>• NO	• YES<br>• NO	• Do not have time to participate in the study<br>• Do not want to participate in a research study<br>• Prefer to test elsewhere

		☐ Able to understand the scope of the study and provide written informed consent<br>☐ Unknown HCV serology status<br>☐ Able to read in Mandarin Chinese<br>☐ Had at least one anal sex episode with another man within the previous 6 months<br>☐ Never used an oral-fluid based self-test			• Prefer not to use self-test<br>• Prefer not to say<br>• Other: ___________________________

Baseline questionnaire

**A. DEMOGRAPHIC DATA OF THE PARTICIPANT**		
**1. STUDY ID:**<br>*HC012-02-0__ __ __*	**2. Date of testing:**<br>*(dd/mmm/yy)*<br><br>………../………………/………..	**3. Full name of the Observer/Interviewer:**<br>______________________________<br><br>**4. STUDY SITE**<br>☐ Guangdong provincial center for Skin and STD control<br><br>☐ Office of Zhitong
**5. Gender:**<br>☐ Male ☐ Transgender	**6. Age:**<br>__________years	**7. Province/City/District (County) of Provenance:**<br>☐ Guangdong/Guangzhou<br>☐ Other: ____________________
**8. Marital status:**<br>☐ Married or living with a partner<br>☐ widow<br>☐ divorced/separated (not living together)<br>☐ Never married	**9. Educational Background:**<br>*Indicate the highest educational level received*<br>☐ Below high school<br>☐ High school (including Zhongzhuan)<br>☐ College (Dazhuan)<br>☐ Undergraduate (bachelor)<br>☐ Postgraduate (master and above)	
**10. How many people live in the household?**<br>Total: ___<br><br>Adults: ____<br><br>Children: ____	11. **Main reason for your visit today?**<br>☐ Screening for hepatitis<br><br>☐ Screening for HIV/syphilis<br><br>☐ Regular health check<br><br>☐Other reason:<br>__________________________<br><br>☐ No answer	**12. Main Occupation:**

B. HEPATITIS C TESTING AND MORBIDITIES

1. **How often do you come to health facilities to check your health?**

☐ More than 1 time per year
☐ 1 time per year
☐ Rarely (once in 2 or more years)
☐ Never

2. Please select all that apply. Have you have received or engaged in

☐ Unprotected anal intercourse
☐ Injecting unprescribed drugs
☐ Sharing needles
☐ A surgical procedure
☐ a dental procedure
☐ Sharing shaving tools or toothbrushes
☐ Make a tattoo

3. **Have you been tested for HCV?**

☐ No, never.
☐ Yes, more than 1 year ago
☐ Yes, in the past 12 months
☐ Do not know
☐ Unwilling to disclose

4. If yes, what was the results of the most recent testing?

☐ Positive ☐ Negative

5. **When was the last time you were tested for HIV? （If you were tested for HIV today, please tell us when was the previous time you were tested）**

☐ I have never been tested for HIV
☐ More than 1 year ago
☐ In the past 12 months
☐ Don't know
☐ Unwilling to disclose

6. If yes, what was the results of the most recent testing?

☐ Positive ☐

7. **Have your partner been tested for HCV?**

☐ No, never.
☐ Yes, more than 1 year ago
☐ Yes, in the past 12 months
☐ Don't know
☐ Unwilling to disclose
☐ No partner

8. If yes, what was the results of the testing?

☐ Positive ☐ Negative
☐ Don't know

9. **Have your partner been tested for HIV?**

☐ No, never.
☐ Yes, more than 1 year ago
☐ Yes, in the past 12 months
☐ Don't know
☐ Unwilling to disclose
☐ No partner

10. If yes, what was the results of the testing?

☐ Positive ☐ Negative
☐ Don't know

11. Do you know that there are some tests that you can do at home to check for medical conditions (e.g. pregnancy test, glucose test)?

☐ No ☐ Yes

12. Have you ever used such a test (self-test) in the past?

☐ No ☐ Yes, blood-based HIV self-test

☐ Yes, other: ______________________

13. If such a test was available to check for hepatitis C, would you be willing to use it?

☐ No ☐ Yes

Additional Observations/Comments:

CHECKLIST ON SELF-TESTING PROCESS

REVIEW DURING THE PROCEEDURE	
**Name of the reviewer:**	
**A. CHECKLIST**	
1. Did the study participant open the pouch and take all of the contents out?	☐ Yes ☐No<br>If no, why: ______________________________
2. Did the study participant read/use the instructions for use before the testing?	☐ Yes ☐No<br>If no, why: ______________________________
3. Did the study participant read/use the instructions for use during the testing?	☐ Yes ☐No<br>If no, why: ______________________________
4. Did the study participant to remove the test tube from the test pack?	☐ Yes ☐No<br>If no, why: ______________________________
5. Did the study participant remove the cap from the test tube?	☐ Yes ☐No<br>If no, why: ______________________________
6. Did the study participant place the test tube in the test stand?	☐ Yes ☐No<br>If no, why: ______________________________
7. Was the study participant able to remove the test device from the test pack?	☐ Yes ☐No<br>If no, why: ______________________________
8. Did the study participant touch the flat pad?	☐ Yes ☐No<br>If yes, why:<br>______________________________
9. Did the study participant collect the sample correctly (1x upper and 1x lower swab of gums)?	☐ Yes ☐No<br>If no, why: ______________________________
10. Did the study participant place the test device in the test tube correctly?	☐ Yes ☐No

	If no, why: ____________________________
11. Did the study participant use a time keeping device (Clock, watch, timer)?	☐ Yes ☐No<br>If no, why: ____________________________
12. Did the study participant read the device results between 20 and 40 minutes after placing the device in the test tube?	☐ Yes ☐No<br>If no, why: ____________________________

B. ADDITIONAL DESCRIPTION DURING THE TEST

1. Mark ALL the problems or mistakes observed when performing the test

☐ Difficulties with opening the pouch
☐ Touched the flat pad
☐ Rubbed the wrong part of the mouth
☐ Rubbed only upper or lower gums and not both
☐ Spilt the fluid from the tube
☐ Poured the fluid from the tube into the stand
☐ Difficulties with sliding the tube into the stand
☐ Test device came out of the tube while testing
☐ Test results read before time
☐ Not able to identify the test line or the control line
☐ Interpreted test results wrongly

☐ No problems or mistakes observed
☐ Any other
Specify:____

2. Which part of self-testing you observed was the most difficult to perform? (Mark one or two)

☐ Opening the pouch
☐ Opening the tube
☐ Sliding the tube into the stand
☐ Placing the test device into the tube
☐ Reading the results
☐ All the steps were easy
☐ All the steps were difficult

3. Was the testing procedure completed?

☐ Yes ☐ No
**4. Was assistance provided? If yes, at which step:**
☐ No<br>☐ Yes, opening the pouch<br>☐ Yes, opening the tube<br>☐ Yes, sliding the tube into the stand<br>☐ Yes, placing the test device into the tube<br>☐ Yes, reminding the test incubation time<br>☐ Yes, reading the results

POST-TESTING QUESTIONNAIRE

Thank you for agreeing to participate today and give your informed consent. I would like to ask you about your experiences of oral self-assessment for HCV self-testing. All your answers will remain confidential and you do not have to answer to questions that you do not want. There are no rights or wrong answers to these questions. Please free to ask questions anytime during the interview and we can stop at any time. Thank you again for your participation.

Acceptability and feasibility of HCV self-testing

Name of the reviewer:

How easy or difficult was it to understand the written instructions for use? ***Read the options***

☐ Very easy ☐ Somewhat easy ☐ Somewhat difficult ☐ Very difficult ☐ Not used

2. How useful were written instruction during the testing procedure? ***Read the options***

☐ Very much ☐ Somewhat ☐ A little ☐ Not at all

3. Thinking about the test, how did you find performing each of these steps: ***Read the options***

	Very difficult	Difficult	Slightly difficult	Slightly easy	Easy	Very easy
**Opening the package**	○	○	○	○	○	○
**Opening the tube**	○	○	○	○	○	○
**Sliding the tube into the stand**	○	○	○	○	○	○
**Swabbing the gums**	○	○	○	○	○	○
**Placing the device into the tube**	○	○	○	○	○	○
**Timing the test**	○	○	○	○	○	○
**Reading the results**	○	○	○	○	○	○

5. Please rate your overall experience with self-test: ***Read the options***

☐ Very easy ☐ Easy ☐ Slightly easy ☐ Slightly difficult ☐ Difficult ☐ Very difficult

6. In your opinion, is HCV ST an accurate test? (Do you trust these results?) *Read the options*
☐ Very accurate ☐ Somewhat accurate ☐ Not accurate ☐ Do not know
**7. Overall, how satisfied were you with the HCV self-testing process?** ***Read the options***
☐ Very satisfied ☐ Somewhat satisfied ☐ A little satisfied ☐ Not satisfied at all
**8. Would you recommend HCV self-testing to a friend or family member?**
☐ Yes ☐ No<br>**Why?**<br>____
**9. How would you feel about taking tests home for your friends or family?**
☐ Positive ☐ Prefer not to ☐ Not sure<br>**Why?**<br>____
**10. Would you like to use this hepatitis C self-test again?**
☐ Yes ☐ No ☐ Not sure<br>____
**11. In your opinion, what are the advantages of self-testing for hepatitis C?**
☐ The test can be performed in privacy ☐ No need to come to a clinic ☐ I can test myself anytime ☐ Not sure ☐ None ☐ Other, specify:
**12. Which are the disadvantages of self-testing for hepatitis C?**
☐ Difficult to perform ☐ No confidence in test results ☐ Getting the results alone (no counselling) ☐ Need to pay for the test ☐ Not sure ☐ None<br>☐ Other, specify:<br>____
**13. What would be your preferred mode to test for Hepatitis C in the future?** ***Read the options***
☐ By myself at home ☐ By myself at a healthcare facility ☐ In a healthcare facility by Health Care Worker ☐ In a screening campaign ☐ Taking a regular sample at a HF ☐ Any, No specific preference ☐ Not sure
**14. Would you prefer a test collecting a blood sample from a fingerstick or no special preference?**
☐ Yes ☐ No ☐ Indifferent
**15. In the case of doing the test by yourself, would you be comfortable on reading any result alone?**
☐ Yes ☐ No ☐ Not sure<br>**Why?**<br>____
**16. What would you do if your HCV self-test is giving a positive result (indicating a likelihood of having hepatitis C infection)?** ***Check all that applies***

☐ Contact health facility ☐ Contact pharmacy ☐ Do a confirmation test (viral load test) ☐ Seek advice from family members and/or friends ☐ Seek advice from a community representative (e.g. NGO representative) ☐ Do not know<br>☐ Other: please specify: ____
**17. Do you know if people can be treated and cured for Hepatitis C?**
☐ Yes, there is a treatment, but not sure about cure ☐ Yes, there is treatment and cure<br>☐ Not sure if treatment or cure ☐ There is no treatment or cure<br>☐ No idea
**18. Do you know if there is treatment available for Hepatitis C in your village/town or near your village/town?**
☐ Yes ☐ Yes, but not nearby ☐ No ☐ Not sure ☐ No idea
**19. Interviewer comments on specific questions, respondent, interview, any additional information**

<u>Thank you for your participation. Do you have any questions you would like to ask?</u>

Supporting Figure S1. Manufacturer's instructions for use (Mandarin Chinese)

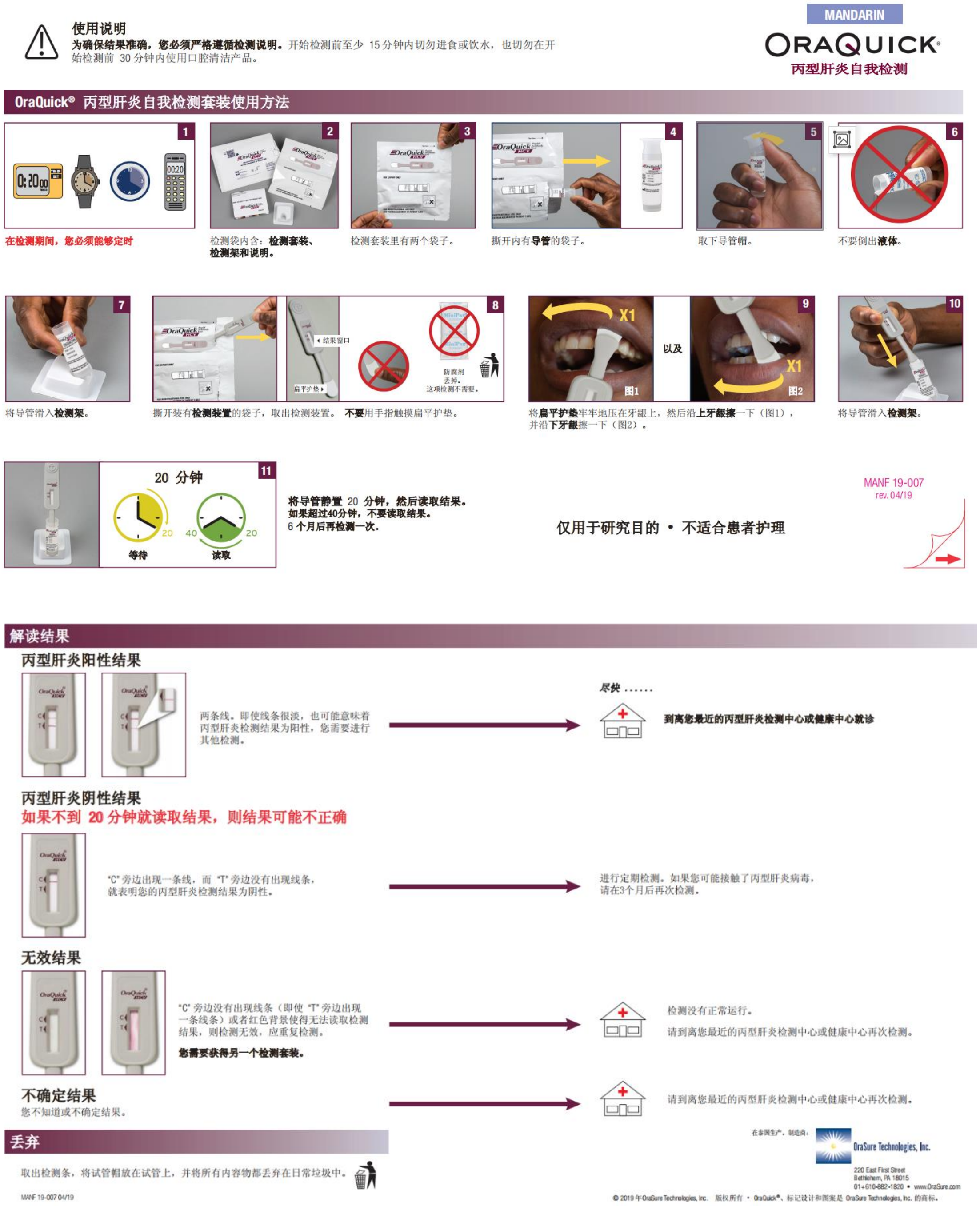

Supporting Table S2. Discordances in results reported by participants and results obtained by re-reading (interpreted by the trained staff) and re-testing (with professional use kit, by the trained staff).

**Study ID**	**Manipulation to collect oral fluid**	**Assistance to read the result**	**Participant's result reading**	**Re-reading**	**Re-testing**	**Observations for discordant results**
HC012020002	Incorrect	No	Negative	Invalid	Negative	Participant swabbed only the upper gums, touched the flat pat, requested assistance with placing the tube into the stand and had an education level below high school.
HC012020405	Correct	Yes	Unsure	Invalid	Negative	Participant had difficulties placing the tube into the stand and requested assistance with reading results.

HC012020410	Correct	No	Unsure	Negative	Negative	Participant poured the buffer liquid into the stand, placed the test device on the stand, read the result after only five minutes and reported the testing process to be somewhat difficult.

Supporting Table S3. Additional Perceptions on HCV Self-Testing

***Overall, how satisfied were you with the HCV self-testing process?* （*N=100*）**	
Very satisfied	63(63.0)
Somewhat satisfied	30(30.0)
A little satisfied	6(6.0)
Not satisfied at all	1(1.0)
Reasons for using HCV self-testing again if available	
Provision of health information by regular testing	27(27.0)
Convenience	17(17.0)
Simplicity	7(7.0)
Privacy	3(3.0)
Reasons for not using HCV self-testing again	
Lack of need	3(3%)
Preference for facility-based testing	2(2%)
lack of knowledge about HCV	1(1%)
Reasons for being unsure about using HCV self-testing again	
Lack of information about the test	5(5.0)
Lack of awareness about risk of HCV infection	2(2.0)
Reasons for recommending the test	
Convenience	30(30.0)
Provision of personal health information	22(22.0)

Privacy	5(5.0)
Reasons for not recommending the test	
Lack of knowledge about HCV	4(4.0)
Lack of need	3(3.0)
Preference for facility-based testing	2(2.0)
Reasons for being unsure about recommending the test	
Lack of confidence in the results	6(6.0)
Lack of knowledge about HCV	2(2.0)
In your opinion, what are the advantages of self-testing for hepatitis C?	
(more than one answer by participant possible)	
Not sure	0(0.0)
None	0(0.0)
The test can be performed in privacy	61(61.0)
No need to come to a clinic	78(78.0)
I can test myself anytime	91(91.0)
Which are the disadvantages of self-testing for hepatitis C?	
(more than one answer by participant possible)	
Difficult to perform	3(3.0)
Not sure	4(4.0)
Need to pay for the test	7(7.0)
No confidence in test results	20(20.0)

Getting the results alone (no counselling)	21(21.0)
None	35(35.0)
In the case of doing the test by yourself, would you be comfortable on reading any result alone? (N=100)	
Yes	96(96.0)
No	4(4.0)
Do you know if there is treatment available for Hepatitis C in your village/near your village? (N=100)	
Yes, nearby	19(19.0)
Yes, but not nearby	1(1.0)
No	2(2.0)
Not sure	21(21.0)
No idea	57(57.0)
